# Coughing and Sneezing: Contact Infections

**Published:** 1918-11

**Authors:** 


### COUGHING AND SNEEZING: CONTACT INFECTIONS

Carriers undoubtedly play an important role in disseminating influenza bacilli, pneumococci, streptococci and meningococci. Men should be specifically instructed at this time against expectorating in quarters, and the dangers of sneezing and coughing, and of speaking in close proximity to the face should be explained.

The handkerchief, or at least the hand, should be held before the mouth when a person coughs. Lieutenant-Colonel Webb has suggested that a "cough drill" be established; that during the regular drills the order "Cough" may be given, and that every soldier of a company be required to cough with his left hand covering his mouth. In this way the soldier may be taught to use this precaution when he has any respiratory infection and is compelled to cough.

In controlling the epidemic of pneumococcic plague in Manchuria, it was found that early detection of cases, with immediate masking of such cases and of doctors and attendants in connection with isolation, were the most important, and indeed indispensable methods of putting down the epidemic. It was shown that in the coughing of the infected cases, the pneumonic plague organism was commonly thrown out into the air of the wards for a distance of from six to eight feet from the mouth. Distance between beds — not cubic space — is the important feature in the prevention of the spread of pneumonia infections. Crowding in recreation rooms, cinema entertainments, etc., should at the present time be prevented as much as possible.

In epidemics of influenza and of pneumonia the disease is undoubtedly usually spread from man to man through the secretions or discharges from the mouth and nose and respiratory tract, and an individual who harbors virulent pneumococci, or streptococci, or influenza bacilli, is obviously very likely to infect his co-sleepers by coughing or sneezing, or even speaking loudly in close proximity to them. Therefore, pneumonia is often a contact disease, and some observers go so far as to say that at least in 80 o/o of the cases the infection arises from the pneumonia patient or a pneumococcus carrier.

It seems evident that, in the majority of instances during the present epidemic of respiratory diseases among our troops in France, its primary infection has spread in barracks, hospitals and billets from contact resulting from overcrowding. In Panama, where the climatic conditions were not severe, pneumonia was prevalent particularly on account of overcrowding, and the same was found to be true among the workers in the South African mines. Prevention consisted particularly in scattering the workmen and giving them separate dwellings in place of barracks. Surgeon-General Gorgas has stated that he was satisfied that the scattering of the individuals was the chief cause of the sudden drop in pneumonia at Panama. Should we not benefit from all this experience?

